# Client satisfaction with cervical cancer screening in Malawi

**DOI:** 10.1186/1472-6963-14-420

**Published:** 2014-09-22

**Authors:** Fresier C Maseko, Maureen L Chirwa, Adamson S Muula

**Affiliations:** Department of Public Health, School of Public Health and Family Medicine, University of Malawi, College of Medicine, Mahatma Gandhi Road, Private Bag 360, Chichiri, Blantyre 3 Malawi; Prime Health Consulting and Services, Prime Health Consulting and Services A47/5/240, Malingunde Road, P.O. Box 1926, Lilongwe, Malawi

**Keywords:** Cervical cancer, Screening, Satisfaction, Visual inspection with acetic acid

## Abstract

**Background:**

Assessing client and patient satisfaction towards a service is of programmatic importance. A study was conducted in Malawi between July and October 2013 to assess client satisfaction among women who had been screened for cervical cancer using Visual Inspection with Acetic acid test.

**Methods:**

This was a cross sectional descriptive study which was conducted in 16 out of 43 cervical cancer screening centres. A semi structured questionnaire was used for data collection. Data were analyzed using STATA version 11 for windows. Descriptive statistics were computed to summarize participant characteristics. Logistic regression was also conducted to assess the relationship between client satisfaction with the service and the independent variables.

**Results:**

One hundred and twenty women with a mean age of 33.7 (SD = 10.1) participated in the survey. All women reported being satisfied with the received service at the facility, with 68.33% reported to be very satisfied. All demographic characteristics such as age, marital status, level of education, with exception of distance to the nearest health facility had no statistically significant association with satisfaction at both univariate and multivariate analysis. However, previous knowledge about the cause of the disease itself, its prevention, knowledge that the disease can be cured, knowledge of clinic times, previous knowledge of the VIA screening test and the source from where they heard about cervical cancer had a statistical significant relationship with the outcome variable. Logistic regression revealed that satisfaction in this study was predicted by having an appointment before the screening with adjusted odd ratio of 5.71(95%CI: 1.75 – 18.63), having previous knowledge of the VIA test, AOR = 0.021(95% CI: 0.002-0.226) distance from the home to the health facility AOR = 0.11(95%CI: 0.02-0.65) and waiting time AOR = 0.09 with 95% CI: 0.09 – 0.83. Having an appointment had the only independent variable with a positive relationship with satisfaction.

**Conclusion:**

Women were satisfied with the screening service. The study also showed several challenges in cervical cancer screening services which can be considered as areas of potential improvement.

**Electronic supplementary material:**

The online version of this article (doi:10.1186/1472-6963-14-420) contains supplementary material, which is available to authorized users.

## Background

Cervical cancer is the most common cancer among women in sub-Saharan Africa. While incidence and mortality rates of cervical cancer have fallen significantly in developed countries, 83% of all new cases that occur annually and 85% of all deaths from the disease occur in developing countries [1]. In Malawi, it is the second most frequent cancer among women between the ages of 15 and 44 years. The incidence is highest among women aged around 40 years [2, 3]. There are 4.50 million women aged 15 and older who are at risk of developing cervical cancer in the country [4]. According to available statistics, cervical cancer was the most common cancer between 2007 and 2010, accounting for 45.4% of all cancer cases in Malawi [5]. Recent evidence suggests an increasing incidence of cervical cancer largely attributed to the high prevalence of HIV and AIDS [3, 6]. Recent statistics from HPV Information Centre estimates that, 3684 Malawian women are diagnosed with cervical cancer every year and 2314 die from the disease [4]. Despite being a fatal disease, screening has been known to reduce cervical cancer morbidity and mortality. Studies have shown that regular screening gives between 80 to 90% reduction in the risk of dying from cervical cancer [7–9].

Malawi has an established cervical cancer prevention program which has been running for more than two decades [10]. In 1999 Project Hope, a non-governmental organization, implemented a comprehensive cervical cancer screening and early treatment project. Initially the project was conducted in the rural district of Mulanje and in the urban district of Blantyre. Later it was rolled out to other districts [11]. Currently, almost all 29 health districts provide cervical cancer screening services using visual inspection with acetic acid (VIA) as recommended by the WHO [10]. Screening cervical cancer with VIA is also known to increase detection of premalignant lesions of the cervix and diminishes the probability of losing women to follow-up before treatment [12]. The screening test provides a single-visit approach in which a health care provider visualizes the women’s cervix using vinegar to detect precancerous cells on the cervix [10, 12]. Sankaranarayanan and his colleagues reported VIA to be feasible, safe, accurate, affordable and an effective means of reducing the cervical cancer burden [13]. Despite the availability of the screening services in most public health facilities in Malawi, statistics shows that very few women have been screened for cervical cancer. The WHO 2010 Malawi summary report indicated that as of the period 2001–02 the cervical cancer screening coverage of all women age 18–69 years, screened every 3 years using different type of cervical cancer screening tests was as low as 2.6%, with 3.7% among urban and 2.5% among rural women [4].

While studies on barriers to the utilization of cervical cancer prevention services have been carried out in Malawi [14, 15], literature on patient satisfaction with the services is scarce. However, conclusions on women’s satisfaction can be made from the experienced barriers such as workers attitudes and unfriendly screening services [14].

Patient satisfaction represents patient’s attitude towards care or its component [16]. It is regarded as a result of an evaluation process (and comparison) of the service [17] obtained from a health care provider and the environment in which care is given. According to Donabedian’s framework for health care evaluation, patient satisfaction is considered a measure of the process of care [18]. It is also related to the extent to which general health care needs and condition-specific needs are met [19]. According to Jackson and his colleagues, patient satisfaction can be used to compare different health programs or systems, evaluate the quality of care, identify which aspects of a service need to be changed to improve patient satisfaction and also to assist health service providers to identify which patients are least likely to continue in a screening or therapeutic program [16].

Satisfaction is an attitudinal response to value judgments that patients make about a clinical encounter [16]. Assessing patient satisfaction towards a service is clinically relevant, as satisfied patients are more likely to comply with treatment, take active role in their own care and continue using services. Where there are some choices, the satisfied patients will stay within a health care provider and maintain within a specific health system [20]. Likewise, patients who are satisfied with their providers’ performance are more likely to continue seeing their care provider, and those who are dissatisfied are more likely to leave [21]. On the part of service providers, knowledge of their patients’ satisfaction is essential to identify areas of improvement in the provision of services. Patient satisfaction studies have shown to have a significant impact on the attitude and behaviours of health care providers and are prone to induce improvement measures in the provision of health care [22]. Convenience of services, which include clinic hours, ability to get an appointment, access to services, and the time spent waiting in the clinic to see the health care provider have been shown to be related to women’ satisfaction with cervical cancer screening services [21, 23].

The present study was conducted in Malawi to assess client/patient satisfaction among women who had been screened for cervical cancer using Visual Inspection with Acetic acid test. Specifically, the aim was to explore patients’ or clients’ perceptions and experiences, as well as to determine their satisfaction with screening.

## Methods

### Design

This was a descriptive cross sectional study which used both qualitative and quantitative approaches. The study was carried out between March and June of 2013.

### Study setting

The study was carried out in 16 public health facilities from 11 randomly selected districts. Malawi is divided into 29 health districts. At the time of the study, according to the statistics obtained from the Reproductive Health unit in the Ministry of Health of Malawi, there were 43 public health facilities nation-wide which were offering cervical cancer screening services using VIA.

### Sampling

The sample was chosen from women who were screened for cervical cancer in the 16 health facilities. The research assistants visited the research facilities during the day and explained the study to the women. Those who gave their consent were invited to participate in the study.

### Data collection

A semi-structured questionnaire, which the Bangladesh National Cervical Cancer Screening Program used in 2008 for a similar exercise as part of their cervical cancer prevention program evaluation [24, 25] was modified to suit the Malawian setting for data collection in this study. Additional file 1 shows the modified questionnaire. The modification took into account the Ware *et al.* determinants of patient satisfaction [26, 27]. In Ware’s framework, patient satisfaction is affected by factors such as interpersonal manner, technical quality, accessibility/convenience, finances, efficacy/outcome, continuity of care, physical environment and availability of care and resources [27]. Following ethical approval in December 2012, a pilot study was conducted in Chikhwawa district to pre-test the drafted tool. The pre-test helped assess the ability of the questionnaire to capture the information needed to achieve the objectives of the study. Information obtained was used to revise some of the questions in the questionnaire. The revised instrument was then used for data collection. Exit interviews were conducted among women who had consented to participate in the study. To avoid social acceptability bias, trained professional nurses, not directly involved in cervical cancer screening interviewed the women who were assured that confidentiality would be respected and were requested to be as honest as possible with their responses.

### Data management and analysis

Completed questionnaires were checked for completeness and consistency by data collection supervisors. A database was created and populated using Epi Info™ 7 (7.1.2) for windows; data entered; transferred into STATA version 11 for window for data cleaning and analysis. Descriptive statistics were computed to summarize participant characteristics. Logistic regression was used to assess the relationship between satisfaction and the independent variables. Satisfaction with cervical cancer screening services was the overall outcome of the analysis. Although the variable was designed to be captured on a 5-point Likert scale from 1(very dissatisfied) to 5 (very satisfied) [28], only two responses (very satisfied and satisfied) were used by respondents making it a dichotomous variable. Each potential independent variable (woman’s age, facility type, distance to the health facility, knowledge of clinic times, waiting time, knowledge of cervical cancer, knowledge that the disease has a cure, source of knowledge of cervical cancer, being counseled before and after the screening) was screened using the Kuskall-Wallis test as appropriate for continuous independent variables and maximum likelihood ratio Chi-square for categorical independent variable [16]. To be certain that Kuskall-Wallis was the right test to use, all potential continuous independent variables were subjected to the Skewness and Kurtosis test to assess the normality of the variables. In the initial binary logistic regression model, satisfaction was regressed on all independent variables that were significant at p < 0.25 during the bivariate analysis The regression models were first subjected to Hosmer and Lemeshow’s (H-L) goodness of fit test to determine whether the fitted model adequately described the observed outcome experience in the data [29]. Odds ratios (ORs) are reported with 95% confidence intervals.

### Ethical consideration

Ethical approval to conduct the study was obtained from the College of Medicine Research and Ethics Committee (COMREC) of the University of Malawi in November 2012. Permission was also obtained from the Ministry of Health Zone Support Offices and District Health offices to conduct the study in the health facilities. To maintain the privacy of the participants, each interview was done in an examination room in the absence of the health service provider. To maintain confidentiality, information obtained from participants was handled only by those authorized.

## Results

One hundred and twenty women with a median age of 32.0, ranging from 15 to 70 years participated in the survey. Twenty four percent of the participants were 25 years old and below, 40% were between 26 and 35 years of age while 36% were 36 years old or older. Most of the women, 84.8%, were married. Almost 73 percent were interviewed from a district hospital. Out of those interviewed at the hospital, 31.4% lived more than 5 km away from their nearest heath facilities. Almost 41% of the women had attended junior primary school education (standard 1–5) while only 5.6% had attended tertiary education. Table 1 below shows detailed socio-demographic characters of the participants.

**Table 1:** **Characteristics of study sample (n = 120)**

Variable	% or median(Range)
**Socio-demographic characteristics**	
**Age (n = 113)**	
Median age Women aged 25 and below	32.0(15, 70)
Women aged between 26 and 35	23.9
Women aged above 35	39.2
**Marital status**	
Married	84.8
Single	7.1
Co-habiting	4.5
Divorced	3.6
**Education**	
Never went to school	29.6
Junior Primary	40.7
Senior Primary	11.1
Junior Secondary	5.6
Senior Secondary	6.5
Tertiary education	6.5
**Type of facility they got screened**	
Central hospital	3.3
District Hospital	72.5
Rural hospital	9.2
Health Centre	15.0
**Distance covered to the facility**	
Less than a Kilometer	31.1
Between 1 and 5 Kilometers	46.2
More than 5 kilometers	22.7
Do you know what causes cervical cancer	
Yes	11.7
No	88.3
Before coming to the health facility did you know that this disease can be cured if detected early?	
Yes	42.5
No	57.5
Before coming to the health facility did you know that this disease can be prevented?	
Yes	35.4
No	64.6
Before you came for screening did you have any knowledge of VIA test?	
Yes	28.2
No	71.8
Before you came for the screening , did you know at what time is the VIA Clinic opened and closed	
Yes	23.3
No	76.7

Most of the respondents had very little knowledge of cervical cancer (Table 1). Only 99 (n = 120) women had heard about cervical cancer. When asked where they first heard about cervical cancer from, almost 35% reported that they had heard about cervical cancer from the health workers, 34% from relatives or neighbors while 30% from the radio. Before coming for the screening test, over 88% of the respondent did not know how the disease is caused. Almost 43% reported to know that the disease could be prevented if detected at an early stage. However 35.4% reported that cervical cancer was preventable and were able to mention at least two risk factors of the disease. Furthermore, 71.8% of the women had no idea of the VIA screening test. When asked if they were aware of when the cervical cancer clinics are opened and closed, less than a quarter (23.3%) knew the times. Despite a few women knowing when the clinics were opened and closed, almost 54% of the women were given appointments on when to come for the screening test.

Some participants, (46.2%), covered a distance of between one and five kilometers to get to the screening centres and travelled on average 69.18(SD = 58.96) minutes. The majority (69.1%) travelled to the health facility in an hour or less. Almost 31% of the participants travelled a distance of less than a kilometer while 23% covered a distance of more than five kilometers to get to the health facilities where they got screened. At the screening centre, 46% of the women waited at least five hours before being attended to. Most of the women (97%) reported being counseled before and after the screening test and all the women except one reported that the examining environment was clean and neat. When asked if their privacy was respected during the whole process of screening, only one woman out of the 120 participants felt that her privacy had not been respected. Seven participants in the survey reported to have received treatment after having been screened. Of the seven, six acknowledged being told of the side effects of the treatment they were given. Almost all the participants except one felt that the health worker who screened them was very good at her work. The participants were also asked if they would accept being referred to a higher hospital in the event of the health worker finding a complication that could not be handled at the facility where they were interviewed. One participant said she would not have accepted going to a higher facility simply because she had no money for transport. Participants were also asked if they would advise their relatives and friends to come for cervical cancer screening at the facility where they were screened. Over 97% of the respondents said they would advise their friends and relatives and three women said that they would not. When asked why they would not, one participant said;

*"…………screening is supposed to be private and why should I go around telling people that I had my private parts shown to a doctor. A woman who cares for her life should know that screening for cervical cancer is good for her life*" (Participant 97). Another participant said "*you know it would be hard for me to convince my friends to come for screening. You know, the Health Surveillance Assistant in our area has been advising women in our village to come for cervical cancer screening, but my fellow women have not taken him seriously. Now if they don’t take, a person who knows more about the diseases seriously, do you think they will listen to me? No I can’t advise them, everyone has her own life to take care of".* (Participant 112)

A five point Likert scale from very good to very bad was used to rate the services received at the health facility; the majority rated it to be very good. There was no statistical significance between rating the service received very good and any of the independent demographic variables. However statistical significant relationships were detected between rating the service very good and previous knowledge that the disease has a cure (*X*^*2*^ = 8.99, P = 0.023), previous knowledge that the disease can be prevented (*X*^*2*^ = 6.65, P = 0.010), and previous knowledge of VIA screening test (*X*^*2*^ = 4.316, P = 0.04).

All women reported at least being satisfied with the service they received at the facility, with 68.3% reported to be very satisfied. Univariate analysis found a statistically significant association between satisfaction and distance to the nearest health facility but not with any demographic characteristics (age, marital status and level of education). However, previous knowledge about the cause of the disease itself, its prevention, knowledge that the disease can be cured, knowledge on clinic times, previous knowledge of the VIA screening test and the source from where they heard about cervical cancer had a statistically significant association with satisfaction. Other variables which had a statistical significant association included, having been given an appointment and waiting time (Table 2).

**Table 2:** **Univariate analyses of demographic variables and satisfaction with the VIA screening service (n = 120)**

Variable	Chi ^2^	d.f	Pvalue
Age	1.377	1	0.2406
Facility Type	5.9325	3	0.115
Marital Status	2.0006	3	0.572
Highest education achieved	3.6421	5	0.602
Distance to the nearest clinic	11.309	1	0.0008
Knowledge of cause of Cervical cancer	7.3447	1	0.007
Previous Knowledge that the disease has a cure	5.9604	1	0.015
Previous Knowledge that the disease can be prevented	15.5375	1	0.00001
Previous Knowledge of VIA Screening test	10.7929	1	0.001
Source of knowledge of Cervical cancer	6.6672	2	0.036
Previous knowledge of Clinic Time	10.1506	1	0.001
Given an appointment for the screening	12.6637	1	0.00001
Counselled before and after the screening	1.9418	1	0.163
Travel time to the facility	1.876	1	0.1708
Waiting time before being screened	7.564	1	0.006
Willingness to advise others to go for screening	0.0028	1	0.958
Did the health worker do a Good work	0.4673	1	0.494
Was the examination room clean and Neat	0.4731	1	0.492
Was their Privacy respected	0.4731	1	0.492
Could they have accepted to be referred to a higher facility	0.9293	1	0.335
How do they rate the services they had received	0.9293	1	0.335

Table 3 shows the final multivariable logistic regression model results. Notably, knowledge of the screening test, appointment, distance travelled to the health facility and waiting time were the only predictors of satisfaction. Appointment was the only predictor with odds ratio greater than one while the other three predictors had odds ratio less than one before and even after adjusting for age and distance to the health facility.

**Table 3:** **Predictors of satisfaction with the VIA screening service resulting from a multivariable logistic regression model adjusted for age and distance (n = 120)**

Variable	***Odds ratio***	***95%CI***		***P-value***
Age				
Women of reproductive age < =49	Referent			
Women above reproductive age >49	1.371534	0.07747	24.2822	0.829
**Distance**				
<1Km	Referent			
1-5Km	0.664387	0.1785	2.47293	0.542
>5Km	0.117849	0.02133	0.65113	0.014
**Waiting time**	0.276422	0.09111	0.83864	0.023
**Appointment**	5.706377	1.74817	18.6268	0.004
**VIA test Knowledge**	0.020976	0.00195	0.22602	0.001

## Discussion

We found no evidence that women’s level of satisfaction with the cervical cancer screening services was associated with their age, marital status and level of education or the type of facility where they accessed the services. However, the level of satisfaction (being very satisfied rather than being satisfied) was higher if they had an appointment, know of the VIA screening test, shorter distance from the place of residence to the health facility and shorter waiting time before being screened. Apart from being married and having primary school education, most of women interviewed were from district hospitals. We believe this was because the national cervical cancer screening program at this time had just been rolled out to all district hospitals and very few districts had started extending the program to their health centres. In some districts, this meant that all cases of cervical cancer were attended at the district hospital. Nevertheless, accessing the screening service at the health centre, community, district or central hospital had no significant relationship with the women’s level of satisfaction.

In almost all health facilities, the setting, in terms of space and equipment was similar. This may also explain why there was no significant difference in satisfaction among the women screened in different types of health facilities. The distance the women travelled to get to the health facilities had a significant contribution towards the women’s satisfaction with the screening services at the health facility. Those women who travelled more than five kilometers had a lower likelihood of being very satisfied with the services than those who travelled five kilometers and less. This finding is consistent with findings from similar studies [30, 31].

There was a significant association between knowledge of the disease in terms of its cause, prevention, cure, screening test and satisfaction in the univariate analysis. However, only knowledge of the VIA screening test had a significant relationship with satisfaction in the multivariate logistic regression analysis. Those women who knew the screening test before coming to the facility were not very satisfied with the screening services compared to those who did not know the screening test earlier. The knowledge the women had, possibly led them to have some expectations of how the screening services would be. It was likely that some of their expectations were not met and as such they could not be very satisfied with the service like those who never knew of the VIA screening test. In this study, knowledge of opening and closing times of clinics was associated with satisfaction in univariate analysis but non-significant in multivariate analysis. Despite not being associated with satisfaction, it can be argued that knowledge of the exact time when the cervical cancer screening clinics were open would enable women to come at the right time for the screening, hence minimizing waiting times before being attended to by the health workers.

There was no association between the time taken to travel from the participant’s dwelling place to the facility and satisfaction with the screening service. However, the time the participants waited before being attended to by the health care provider had a significant relationship with satisfaction with the cervical cancer screening service. Women who waited longer were not more satisfied than those who waited shorter regardless of the type of health facility they went to. This is consistent with previous studies which have shown a negative correlation between the time spent waiting before being examined by a health care provider and satisfaction with services received [30, 32–34]. The more time the women spent waiting for the service, the less the satisfaction with the service [21, 33, 35]. The shortest waiting time was three hours. This may suggest the need to identify inefficiencies in the way cervical cancer prevention services are delivered. Regardless of the time they spent waiting, the majority of women were very satisfied with the services. This is the case where services are of low cost or free. A study designed to assess patients’ satisfaction regarding outpatient services at Dhaka Medical College Hospital in Bangladesh, where the cost of treatment was free and/or low, patient expressed satisfaction despite long waiting time [36].

Being given an appointment had an impact on the satisfaction of the participants. Those given appointments and were screened on the day of their appointment were more likely to be very satisfied than those who were not. The majority of health facilities that participated in this study did not conduct cervical cancer screening clinics on daily basis. Most health facilities had cervical cancer screening clinics once or twice a week due to lack of resources or personnel. So, being screened on the day of appointment increased the satisfaction with the services. Almost every participant reported that their privacy during the screening was respected. This is not surprising since in all the health facilities screening service were offered in closed rooms and the screening was done on an individual basis.

There was no significant difference in the level of satisfaction between those who were counseled before and after the screening procedure and those who were not. No significant difference was also observed between those who perceived that the health care provider demonstrated a great deal of professionalism and competence during the whole process of screening and those who perceived that the examination room was clean and neat or not. This is consistent with similar studies which assessed satisfaction of women with health care services [21]. However, it should be noted that in this study, over 80 percent of the participants were women who either did not have a formal education or had a primary education only. With this low educational status, it is very unlikely that these women knew any screening procedures and guidelines to understand the importance of the pre and post counseling in cervical cancer screening. Although they did not have the clinical expertise to critically assess the competencies of the health care providers, it is possible that their judgment of professionalism and competence was merely based on the health care providers’ personality. In a recent study conducted by Kumbani and her colleagues in which they wanted to find out if Malawian women critically assess the quality of care, the researchers found that women’s satisfaction with care did not essentially mean that the quality of the care they had received was good but rather indicated low or no expectations [37]. This could hold true in a country where the majority of the women do not know their health rights [38] and where the health care services are considered to be of low quality [37]. We noted that women in this study were very satisfied with the services although the demand for the cervical cancer screening is very low in Malawi. This could be explained by the poor knowledge that women have about the cervical cancer. As the findings show, not all the participants knew about cervical cancer before having the screening test. This is not surprising as previous studies done in Malawi have showed the same results [10, 11, 15]. In the absence of adequate knowledge, women are likely not to present for cervical cancer screening [14].

### Limitations

The study has several limitations. Firstly, exit interviews were conducted only for a day at each participating health facility. Most of the clinics had more than one trained and active providers who were conducting the screening clinics in turns. Since the research assistant had only one day to conduct the interviews, it was possible for facility managers to allocate the best health care provider to do the screenings on the interview day. Secondly, patient satisfaction surveys are prone to response bias due to social desirability that can be influenced by the interviewer’s characteristics and attitudes. Considering the fact that the interviews were face-to-face interviews and that most of the participants were of low education status, the possibility of this bias cannot be ruled out. Despite the districts participating in the study being randomly selected, and all health facilities in the districts included in the sample, participants were not randomly chosen. Thus it would be difficult to generalize the findings.

## Conclusion

Our findings indicate that all participants were satisfied with cervical cancer screening using visual inspection with acetic acid. Higher satisfaction levels were independent of their socio-demographic characteristics, the type of facility attended for screening and having poor or little knowledge of cervical cancer. The study also revealed several challenges in cervical cancer screening services which can be considered as areas of possible improvement. These areas include long waiting time, long distance to the screening centres and lack of knowledge of the disease among the participants. The findings can be used to inform cervical cancer prevention program managers at both policy and implementation levels what need to be done to improve cervical cancer screening and coverage in the country.

## Electronic supplementary material

Additional file 1: **Client satisfaction survey questionnaire.** (DOCX 42 KB)
