# Supplementary material for: Client satisfaction with cervical cancer screening in Malawi
Source: BMC Health Serv Res. 2014 Sep 22;14:420. doi: 10.1186/1472-6963-14-420 (PMC4180310; doi:10.1186/1472-6963-14-420)
Supplement: Supplementary file 1 — Additional file 1: Client satisfaction survey questionnaire. (DOCX 42 KB) [file 12913_2014_3509_MOESM1_ESM.docx]

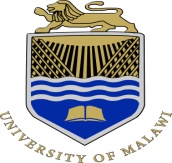


**University of Malawi**

**College of Medicine**

**Data collection tool for client satisfaction survey**

1. **Identification of respondent**

Interviewer name: …………………………………………Date of Interview: …. /…………/2012

Facility name:……………………………………………District……………………………………. Age……………….

Type of facility: *(tick the appropriate choice)*

- - - - Central hospital
      - District hospital
      - Rural hospital
      - Health Centre
      - CHAM
      - Private

Marital status: *(tick the appropriate choice)*

- Single
- Married
- Co-habiting
- Divorced
- Widowed

**`**

Highest education level attained

- Junior Primary
- Senior primary
- Junior Secondary
- Senior Secondary
- University

1. **Access to health care**
   1. Where do you usually go for treatment if you fall sick? ………………………………………………………
   2. How far is the nearest hospital/health center from your house? ____ Km/Unknown
2. **Knowledge about cervical cancer and its prevention?**
   1. Before you came for the screening, did you have any knowledge of cervical cancer

- Yes
- No
  1. Could you please describe what you understand about the disease?

Cause: …………………………………………………………………………………………………………………………………………………………………………………………………………………………………………………………………..

Risk factors: ……………………………………………………………………………………………………………………………………………………………………………………………………………………………………………………………………

- 1. Before coming to the health facility did you know that this disease can be prevented?
- Yes
- No
  1. If yes, did you know how it can be prevented?
- Yes
- No
  1. Before you came for screening did you have any knowledge of VIA test?
- Yes
- No
  1. If yes, where did you hear it from?
- Health workers
- Relative/neighbors
- Billboard/poster
- Radio
- Television
- Newspapers

1. **Knowledge about the VIA services.**
   1. Do you know the time when VIA clinic is opened and closed?

- Yes
- No
  1. Were you given an appointment when you were to be tested?
- Yes
- No
  1. Can you please tell me what it means by negative test result? ………………………………………………………………………………………………………………………………………………………………………………………………………………………………………………………………………………………………………………………………………………………………………………………
  2. Can you please tell me what you understand by positive test result? …………………………………………………………………………………………………………………………………………………………………………………………………………………………………………………………………………………………………………………............................................................................................................

*(Question 4.5 and 4.6) to be answered by those tested positive)*

- 1. If you tested positive, have you been given treatment?
- Yes
- No
  1. Have you been told the side effects of the treatment you have been given
- Yes
- No

1. **Experience after going through the test**
   1. Do you think the health worker who tested you was good at his/her work?

- Yes
- No
  1. Anything you think could be improved? ..................................................................................................................................................................................................................................................................................................................................................................................................................................................................................................................
  2. Was the examination center neat and clean?
- Yes
- No
  1. Was your privacy during examination adequately maintained?
- Yes
- No
  1. Did the nurse explain to you before and after the test?
- Yes
- No
  1. How much time did you spend to travel from your home to the center? …hrs.
  2. How much time did you have to wait before the test at the center? …………hrs.
  3. How can you rate the overall experience you had at the center?
- Very bad
- Bad
- Neither good or bad
- Good, could be better
- Good
- Very good
  1. Any suggestions which you think might improve the service? ………………………………………………………………………………………………………………………………………………………………………………………………………………………………………………………………………………………………………………………………………………………………………………………
  2. Will you advice your friend/relatives to go for the VIA test?
- Yes
- No
  1. If you were to be referred to a higher health facility could you have gone there?
- Yes
- No
  1. If not, why couldn’t you have gone? ……………………………………………………………………………………………………………………………………………………………………………………………………………………………………………………………………………………………………………………………………………………………………………………………………………………………………………………………………………………………………………………
  2. In conclusion, how satisfied were you with the cervical cancer prevention services at this facility?
- Very satisfied
- Satisfied
- Nether satisfied or not
- Not Satisfied
- Very unsatisfied
